# Machine Learning-Based Prediction of Fetal Macrosomia Using Maternal: A Pilot Study

**DOI:** 10.3390/diagnostics16162661

**Published:** 2026-08-20

**Authors:** Tuğba Tahta, Zafer Bütün, Özer Çelik, Ece Akça Salik, Yeliz Kaya

**Affiliations:** 1 Midwifery Department, Faculty of Health Sciences, Ankara Medipol University, Ankara 06050, Turkey; 2 Hoşnudiye Mah. Ayşen Sokak Dorya Rezidans, Ablok no:28/77, Eskişehir 26130, Turkey; 3 Vocational School of Information Technologies, Anadolu University, Eskişehir 26470, Turkey; 4 Department of Gynecology and Obstetrics, Eskisehir City Hospital, Eskişehir 26080, Turkey; 5 Department of Gynecology and Obstetrics Nursing, Faculty of Health Sciences, Eskişehir Osmangazi University, Eskişehir 26040, Turkey

**Keywords:** macrosomia, machine learning, perinatal health, early risk assessment

## Abstract

**Objective:** This study aimed to develop machine learning (ML)-based models for the early prediction of macrosomia using only maternal sociodemographic and obstetric data. **Methods:** This retrospective study included 100 pregnant women who delivered at the Obstetric Clinic of Eskisehir City Hospital between January 2022 and December 2023. Participants were classified as nulliparous (*n* = 48) or parous (*n* = 52) and further categorized according to the presence or absence of fetal macrosomia (birth weight > 4000 g). Predictor variables included maternal age, body mass index (BMI), gravida, smoking status, history of diabetes mellitus, and hypertension; previous birth weight was additionally included in the parous model. Separate machine learning models were developed for nulliparous and parous women using Extra Trees Classifier, Light Gradient Boosting Machine (LGBM), eXtreme Gradient Boosting (XGB) Classifier, Random Forest, and Logistic Regression. The dataset was randomly divided into training (80%) and testing (20%) subsets. Internal validation was performed using 10-fold cross-validation within the training dataset to optimize model performance and reduce overfitting. Given the relatively small sample size, this study was designed as a pilot exploratory investigation. **Results:** Overall, 48 nulliparous and 52 parous mothers were included in the study. Among the nulliparous women, 22 (45.8%) had macrosomic newborns, whereas 26 (54.2%) had normal birthweight newborns. Among parous women, 28 (53.8%) had macrosomic newborns, while 24 (46.2%) had normal birthweight newborns. For nulliparous mothers, the XGB Classifier achieved the highest accuracy (80%) and AUC-ROC (82.2%), demonstrating robust predictive performance. For parous mothers, the XGB Classifier again outperformed other ML models, achieving an accuracy of 72.7% and an AUC-ROC of 83.3% in predicting macrosomia. **Conclusions**: This study highlights the feasibility of ML-based decision support systems in obstetrics, particularly in low-resource settings, to predict macrosomia using readily available maternal characteristics. As a pilot study, these findings should be interpreted cautiously and require validation in larger multicenter cohorts before clinical implementation.

## 1. Introduction

Macrosomia, one of the most significant adverse perinatal outcomes, is commonly defined as a birth weight exceeding 4000 g [1,2]. This pregnancy complication is associated with severe maternal and neonatal morbidity, including shoulder dystocia, brachial plexus injury, asphyxia, prolonged labor, postpartum hemorrhage, and anal sphincter laceration [1,3]. Additionally, macrosomia increases the likelihood of cesarean delivery and neonatal metabolic complications, contributing to long-term health risks for both mother and child. Given these potential complications, early identification of macrosomia is of paramount importance for optimizing perinatal care. The accurate prediction of this condition could allow for better delivery planning, including preparing the operating theatre for emergency interventions and neonatal intensive care unit readiness before birth. Several maternal and fetal risk factors have been associated with macrosomia. Among these, pre-pregnancy body mass index (BMI) [4,5,6,7,8,9], gestational diabetes mellitus (GDM) [10], gestational weight gain [4,5,8], male fetal sex [4,5], parity [4], advanced maternal age [4,5], neck and waist circumference [11], and a previous history of macrosomia [7,8] have been extensively studied as key predictors. Traditional nomogram models incorporating these variables have been developed to estimate the risk of macrosomia [12,13,14,15,16,17].

In recent years, machine learning (ML) methods have been increasingly applied across various medical disciplines to improve risk prediction, prognostic assessment, and clinical decision-making. Beyond fetal macrosomia, recent studies have also demonstrated the utility of machine learning for predicting other obstetric complications, including shoulder dystocia, using maternal and fetal biometric characteristics [18]. For example, ML-based models have been proposed for predicting postoperative complications following general surgery [19], emergency cesarean section among nulliparous women [20], preterm birth [21], neonatal intensive care unit admission [22], and gestational diabetes mellitus [23]. In addition, systematic reviews have reported promising results regarding the application of ML algorithms in predicting postpartum hemorrhage [24] and renal cancer recurrence [25]. These studies demonstrate that ML approaches can effectively identify complex interactions among clinical variables and provide clinically meaningful predictive performance across diverse healthcare settings. Collectively, these findings highlight the growing role of artificial intelligence in healthcare and support the exploration of ML-based approaches for predicting adverse perinatal outcomes, including fetal macrosomia.

Compared with conventional statistical approaches, machine learning algorithms offer several potential advantages for clinical prediction tasks. Traditional statistical models generally require predefined assumptions regarding the relationships between predictors and outcomes and may have limited ability to capture complex nonlinear interactions among variables. In contrast, machine learning methods can automatically identify hidden patterns and interactions within multidimensional datasets, potentially improving predictive performance and model adaptability [23,24,25]. Given that fetal macrosomia is influenced by multiple interacting maternal characteristics and metabolic factors, machine learning approaches may provide a useful framework for developing more flexible and accurate prediction models using routinely collected clinical data.

In recent years, there has been a rapid expansion in the use of artificial intelligence (AI) approaches in obstetrics, particularly for predicting perinatal complications such as macrosomia [26]. More recently, Liu et al. [27] demonstrated that machine learning models developed using data collected at different stages of pregnancy, including the pre-pregnancy, first-trimester, and pre-labor periods, achieved promising predictive performance for fetal macrosomia. Similarly, Chen et al. [28] developed and externally validated machine learning models for macrosomia prediction using multidimensional clinical data, further supporting the growing role of artificial intelligence in obstetric risk prediction. (ML)-based predictive models have been constructed as clinical decision support systems to enhance early detection and risk stratification of macrosomia [29,30,31,32,33,34]. However, most existing ML models rely heavily on biochemical markers and ultrasound measurements to achieve accurate predictions [30,31,32,33,34]. Recent studies have also incorporated extensive laboratory parameters, fetal biometric measurements, and multidimensional clinical variables to improve predictive accuracy, although these approaches may limit applicability in routine or resource-limited antenatal care settings [27,28,35] While these approaches are valuable, they may not always be accessible, particularly in resource-limited settings or in routine antenatal care where ultrasound evaluations and advanced biomarkers are not systematically available.

To address this gap, we aimed to develop and internally validate machine learning models for predicting fetal macrosomia using only routinely available maternal sociodemographic and obstetric characteristics. By intentionally excluding ultrasound measurements and biochemical markers, we sought to evaluate whether acceptable predictive performance could be achieved using information readily accessible during routine antenatal care. This approach may facilitate the development of scalable and cost-effective decision-support tools, particularly in resource-constrained healthcare settings. We hypothesized that machine learning models based solely on routinely available maternal sociodemographic and obstetric characteristics would achieve acceptable predictive performance for fetal macrosomia without requiring ultrasound or biochemical variables.

## 2. Materials and Methods

### 2.1. Study Design and Population

This retrospective observational pilot study was designed to develop and internally validate machine learning models for predicting fetal macrosomia. We collected the maternal and pregnancy-related data of the patients who were admitted to the Eskisehir City Hospital Obstetric Clinic between 1 January 2022 and 31 December 2023 retrospectively after ethical approval had been obtained. Informed consent was not obtained from participants due to the retrospective study design.

We identified the inclusion criteria as:

- Having hospital admittance in the first trimester of pregnancy.
- Being >18 and <40 years old.
- Having spontaneous, non-anomalous, and singleton pregnancy.
- Having all data for the sociodemographic information of the pre-conceptional period (age, BMI, history of diabetes mellitus, and hypertension), obstetrics history (gravida, parity, and previous birth weight), and the newborn’s birth weight.
- Having a maximum of two live births.

### 2.2. Measures

The maternal age, Body Mass Index (BMI), gravida, parity, previous birth weight, smoking status, history of diabetes mellitus, and hypertension were collected from the electronic hospital records retrospectively.

Fetal macrosomia was defined as a birth weight of >4000 g. After determining the participants of the study with the inclusion criteria, all mothers were grouped as macrosomic and non-macrosomic according to the newborn birth weight. The study population was intentionally constructed to include comparable numbers of macrosomic and non-macrosomic pregnancies to facilitate supervised machine learning model development rather than to estimate the prevalence of fetal macrosomia. Consequently, the observed proportion of macrosomia in the study cohort does not reflect its prevalence in the general obstetric population and should not be interpreted as an epidemiological estimate. For further stratification, participants were also classified into subgroups as nulliparous (pregnant women who had never given birth before the current pregnancy) and parous (pregnant women who had previous live births) to refine the predictive modeling process by parity. Stillbirths were not included in the classification criteria.

This study aimed to develop two separate machine learning (ML) models for predicting macrosomia in nulliparous and parous pregnant women, as combining all cases into a single dataset would have led to missing data issues for variables, such as the history of gestational diabetes mellitus (GDM) and previous birth weight, which are only applicable to parous women. The dataset underwent several preprocessing steps to enhance data quality and ensure optimal model performance. Missing values accounted for less than X% of the dataset. To minimize information loss, missing values were imputed using the training dataset only. Continuous variables were imputed using the mean, categorical variables using the mode, and model-based imputation was applied when complex variable interactions were identified.

To construct robust predictive models, various ML algorithms were employed, including the Extra Trees Classifier, Light Gradient Boosting Machine (LGBM), eXtreme Gradient Boosting (XGB), Random Forest, Logistic Regression. Ensemble methods, such as Extra Trees, LGBM, and Random Forest, were selected for their ability to capture non-linear relationships and interactions within the dataset, while logistic regression was included due to its interpretability and clinical relevance.

To develop and evaluate the machine learning models, the dataset was randomly partitioned into training (80%) and testing (20%) subsets using stratified sampling to preserve the proportion of macrosomic and non-macrosomic cases in both datasets. Model development was performed exclusively on the training dataset. Internal validation was conducted using 10-fold cross-validation within the training set, whereby the training data were divided into ten approximately equal folds; in each iteration, nine folds were used for model training and one fold for validation. This procedure was repeated until each fold had served once as the validation set, and performance estimates were averaged across folds. The independent test dataset remained completely isolated during model development and cross-validation procedures and was used only once for final model evaluation.

Each model’s performance was assessed using key evaluation metrics, including accuracy, sensitivity (recall), specificity, F1-score, and the area under the receiver operating characteristic (ROC) curve (AUC). A confusion matrix was analyzed to gain insights into classification errors by identifying false positives and false negatives. The final model was selected based on its overall performance across multiple evaluation metrics rather than ROC-AUC alone. Model selection considered the balance between ROC-AUC, sensitivity, specificity, accuracy, F1-score, and clinical interpretability. Minimizing false positives was considered important to reduce unnecessary interventions and maternal anxiety, whereas minimizing false negatives was essential to improve the identification of pregnancies at risk of fetal macrosomia and support appropriate perinatal management. By developing separate models for nulliparous and parous women, this study accounted for differences in pregnancy history, aiming to improve the clinical applicability and reliability of ML-based macrosomia prediction.

To determine the most significant predictors of macrosomia, the Permutation Feature Importance method was applied. This model-agnostic technique evaluates the importance of each independent variable by measuring the decline in model performance when the values of that variable are randomly shuffled. A greater drop in model accuracy indicates a higher dependence on that variable, making this method valuable for identifying key risk factors while maintaining model interpretability.

**Figure 1:**
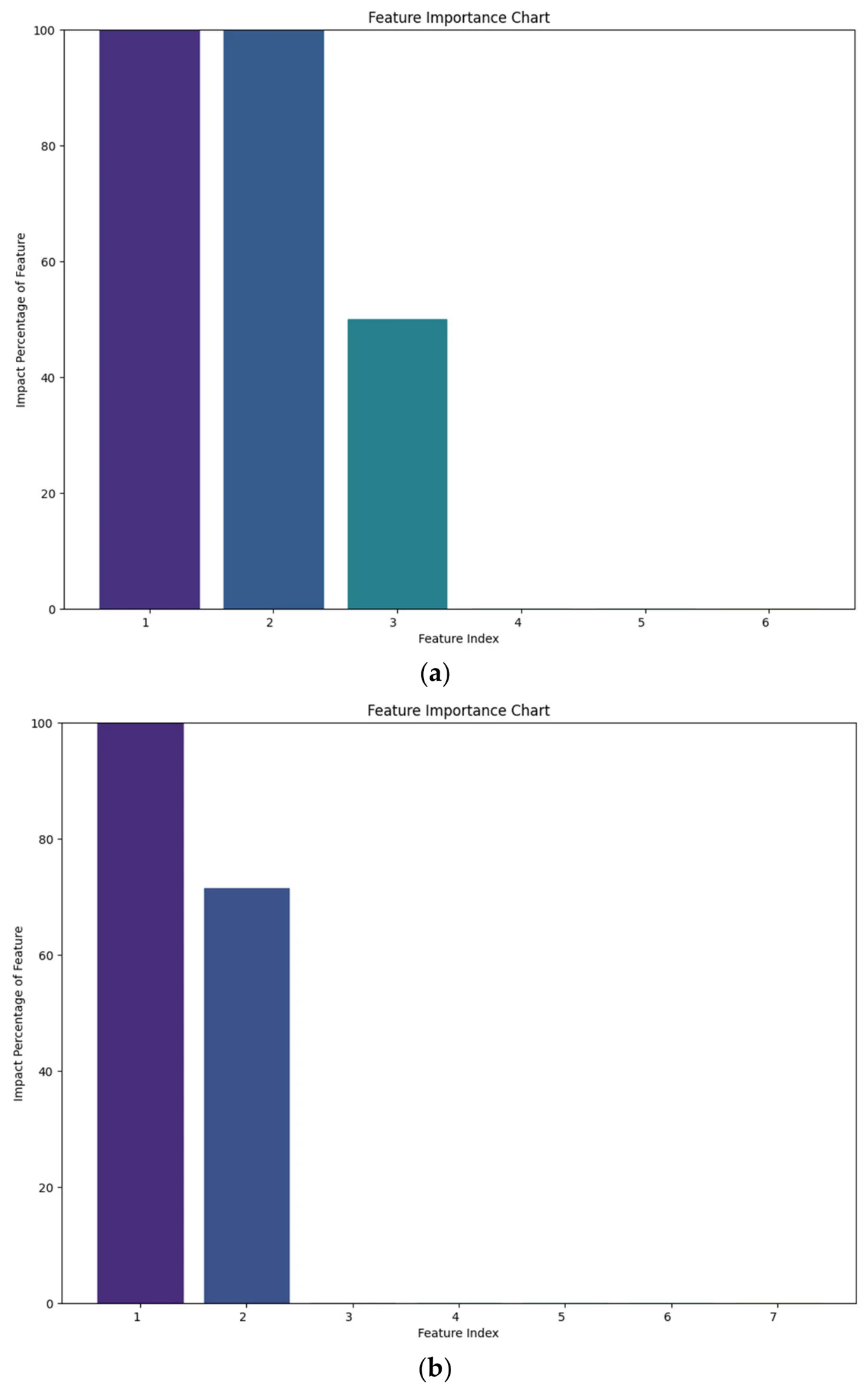
(**a**) Feature Importance Chart of the variables for the primiparous mothers’ group. 1: the history of diabetes mellitus, 2: Body Mass Index, 3: Age. (**b**) Feature Importance Charts of the variables for the multiparous mothers’ group. 1: Age, 2: Body Mass Index.

For nulliparous women, the most influential predictors identified were the history of diabetes mellitus, body mass index (BMI), and maternal age, while for parous women, the most critical predictors were maternal age and BMI. These findings highlight the role of metabolic and maternal factors in macrosomia risk across different pregnancy histories. The Permutation Feature Importance Plot (Figure 1a,b) illustrates the relative contributions of these variables, providing a transparent ranking of feature significance. 

This approach not only enhances model interpretability but also offers valuable insights for clinical decision-making by emphasizing the most impactful factors in macrosomia prediction.

Predictors included in ML models;

Nulliparous model

- Maternal age;
- BMI;
- Gravida;
- Smoking status;
- Hypertension history;
- Diabetes mellitus history.

Parous model

- Maternal age;
- BMI;
- Gravida;
- Smoking status;
- Hypertension history;
- Diabetes mellitus history;
- Previous birth weight.

The predictor variables were selected based on established clinical risk factors for fetal macrosomia reported in previous studies and their routine availability during antenatal care. Maternal age, pre-pregnancy body mass index (BMI), smoking status, history of hypertension, and diabetes mellitus were available for both nulliparous and parous women. Previous birth weight was included only in the parous model because this variable is inherently unavailable in first pregnancies. Maternal BMI was calculated from pre-pregnancy weight and height recorded at the first antenatal visit. Diabetes mellitus included pre-existing diabetes or gestational diabetes mellitus when documented in the medical records.

Several precautions were taken to minimize the risk of data leakage. The dataset was first partitioned into training and testing subsets prior to model evaluation. The independent test set was not accessed during model development, feature importance assessment, cross-validation, or model selection procedures. Missing value imputation was performed separately within the training and testing datasets to prevent information transfer between datasets. No outcome-derived variables were included among the predictors. Hyperparameter optimization was not performed because of the limited sample size and the pilot nature of the study; therefore, default algorithm settings were used across models. The primary objective was to compare algorithm performance under identical conditions while minimizing the risk of overfitting. No automated feature selection procedure was applied. All clinically relevant variables available in the dataset were entered into the machine learning algorithms.

### 2.3. Data Analysis

The Shapiro–Wilk test was used to test the normality of the variables in the analysis. Continuous variables were expressed as mean ± standard deviation, and categorical variables as frequency (percentage) and median (minimum–maximum). The comparison of continuous variables that showed normal distribution between groups was made using the independent samples t-test; however, the Mann–Whitney U test was used for the comparison of continuous variables that did not show normal distribution. The relationship between categorical variables between the groups was analyzed using the Chi-Square test. In all analyses, a two-sided *p*-value < 0.05 was considered statistically significant. All statistical analyses were performed using IBM SPSS for Windows version 15.0, while ML algorithm tests were conducted with Python software (Python Software Foundation, Python Language Reference version 3.5). Machine learning analyses were performed using Python version 3.5. The primary libraries included scikit-learn, XGBoost, and LightGBM. Because of the pilot nature of the study and limited sample size, hyperparameter optimization was intentionally not performed, and default algorithm settings were used. The objective was to compare model performance under standardized conditions while minimizing the risk of overfitting.

## 3. Results

The demographic and obstetric characteristics of the 100 mothers included in the study, classified as nulliparous and parous, and further categorized into macrosomic and non-macrosomic newborn groups, are presented in Table 1. Among nulliparous mothers, 22 (45.8%) had macrosomic newborns, while 26 (54.2%) had newborns with normal birth weight. Among parous mothers, 28 (53.8%) had macrosomic newborns, while 24 (46.2%) had normal birth weight newborns.

**Figure 2:**
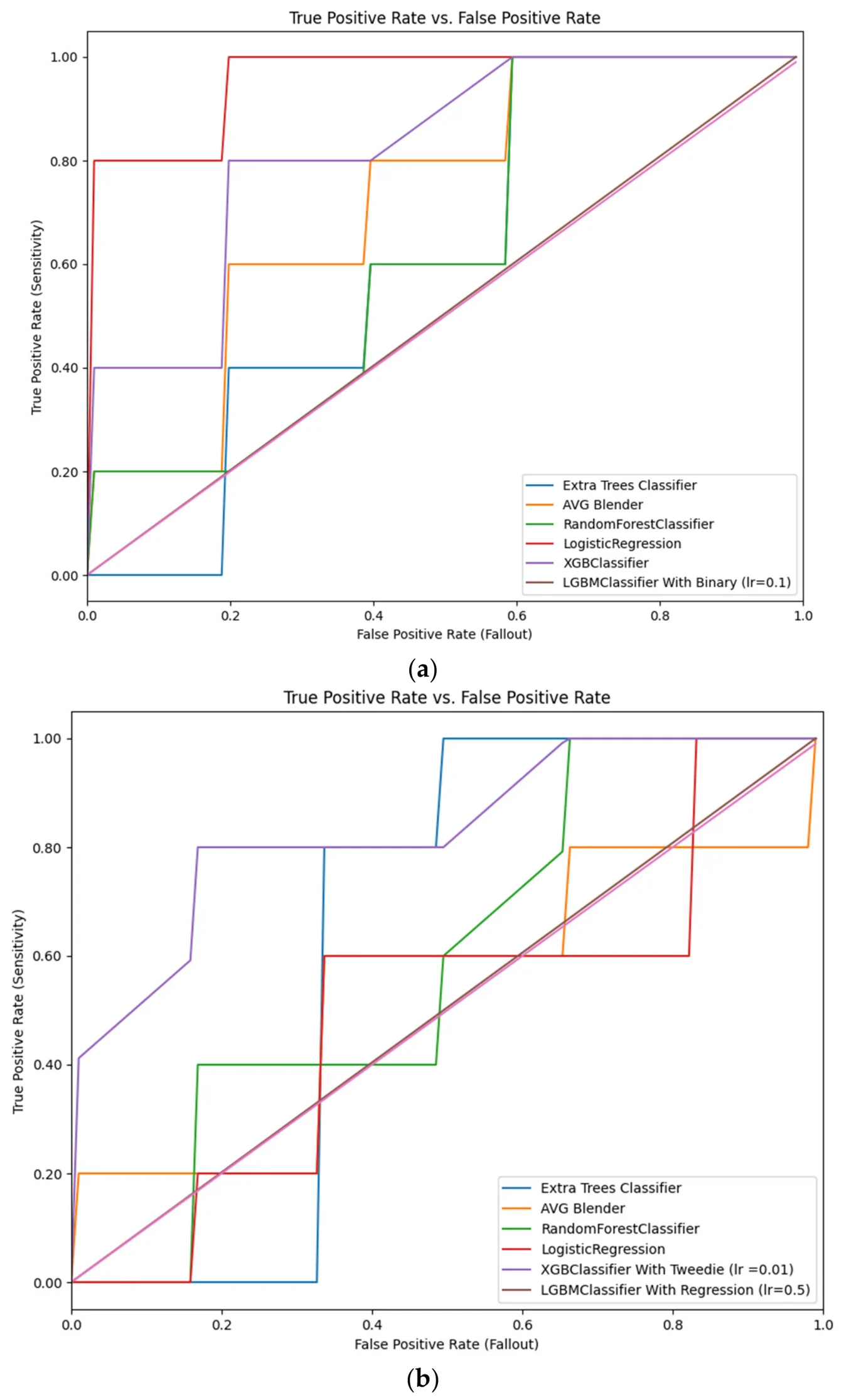
(**a**) Receiver operating characteristic curve graphs of the primiparous mothers’ variables. (**b**) Receiver operating characteristic curve graphs of the multiparous mothers’ variables.

In the first part of the study, statistical analysis of nulliparous mothers revealed that the history of diabetes mellitus and maternal BMI were significantly different between those who had macrosomic and normal birthweight newborns (*p* = 0.002 and *p* = 0.001, respectively) (Table 1). The Permutation Feature Importance method identified the history of diabetes mellitus, BMI, and maternal age as the most important predictors of macrosomia (Figure 1a). Following model training, the ML algorithms were applied to the test dataset, and the XGB Classifier emerged as the most effective model, achieving an accuracy of 80% and AUC-ROC of 82% (Table 2, Figure 2a). Both sensitivity and specificity were 80% (Table 3), indicating a well-balanced model performance.

In the second part of the study, statistical analysis of parous mothers showed no statistically significant differences between the macrosomic and normal birthweight groups (Table 1). However, the Permutation Feature Importance method identified maternal age and BMI as the key predictors of macrosomia (Figure 1b). Again, after training and testing the ML models, the XGB Classifier demonstrated the highest predictive performance with an accuracy of 72.7% and AUC-ROC of 83.3% (Table 2, Figure 2b). In this cohort, sensitivity was 60%, while specificity was 83.3% (Table 3), suggesting that while the model effectively identified non-macrosomic cases; however, its sensitivity in detecting macrosomia was slightly lower compared to the nulliparous model.

## 4. Discussion

Macrosomia is a well-documented risk factor for both maternal and neonatal morbidity and mortality, emphasizing the need for its early and accurate prediction during pregnancy. Several risk factors, including GDM, pre-pregnancy BMI, and maternal age, have been associated with macrosomia, and traditional nomogram-based models have been developed to estimate its likelihood. While these nomograms are relatively simple and easily applicable in daily obstetric practice, they have notable limitations. Most have been developed using traditional statistical methods and often focus on pregnant women with GDM rather than the general obstetric population [12,14]. Furthermore, while sociodemographic and obstetric characteristics are frequently included, many nomograms also rely on biochemical laboratory results and ultrasound measurements, which may not always be available, particularly in low-resource settings [13,16,30]. Importantly, none of the previously published nomograms have undergone external validation, raising concerns about their real-world predictive performance.

Access to healthcare remains a significant barrier for many pregnant women worldwide. According to the World Health Organization, at least four antenatal visits are recommended during pregnancy. However, nearly 75% of pregnant women in some regions, particularly in Africa, do not receive the minimum required antenatal care [36]. Given these disparities, there is a growing need for alternative, easily accessible risk assessment tools that do not depend on advanced medical equipment or highly specialized personnel. ML-based clinical decision support systems could serve as valuable tools for predicting pregnancy-related complications, including macrosomia, especially for populations with limited access to comprehensive obstetric care. Unlike traditional statistical models, ML methods offer advantages such as the ability to identify complex, non-linear relationships, integrate multiple predictors, and enhance predictive accuracy. Previous studies, including those by Shigemi et al. [33] and Wang et al. [29], have demonstrated that ML-based macrosomia prediction models can outperform logistic regression in terms of sensitivity, specificity, and overall classification performance.

Shigemi et al. [33] were among the first researchers to develop a predictive ML model for macrosomia, using maternal physical parameters across all pregnancy trimesters by applying a random forest algorithm, achieving an AUC of 0.866. Similarly, Wang et al. [29] incorporated pre-pregnancy BMI, maternal age, number of pregnancies, parity, and additional pelvic measurements at 12 weeks of gestation to predict macrosomia, reporting a sensitivity, specificity, and AUC of 91.7%, 91.7%, and 95.3%, respectively, for their random forest model. In another study, Liu et al. [31] utilized various ML techniques to identify key predictors of infant birth weight, including maternal height and pre-pregnancy BMI, although their study did not provide a detailed interpretation of model performance. Kang et al. [34] focused specifically on macrosomia prediction in women with GDM, employing artificial neural networks, decision trees, and support vector machines, with neural networks demonstrating the best performance. Ye et al. [32] developed a multi-step ensemble learning approach incorporating prenatal ultrasound measurements. Liu et al. [30] introduced a deep learning-based framework with recurrent neural networks, combining data augmentation and longitudinal learning to enhance prediction accuracy. More recently, Liu et al. [27] reported that logistic regression achieved the best predictive performance during the pre-pregnancy and first-trimester periods, whereas an ensemble model performed best before labor, emphasizing that predictive performance varies according to the timing of data acquisition. Likewise, Cui et al. [35] demonstrated excellent discrimination using interpretable XGBoost models and highlighted the importance of metabolic and biometric maternal factors for macrosomia prediction. These findings are consistent with our observation that maternal BMI and diabetes-related variables contributed substantially to prediction performance.

Despite these studies, none of the existing ML models have fully addressed the key requirements for an ideal macrosomia prediction tool. Shigemi et al. [33] and Kang et al. [34] relied on pregnancy-period data, which may limit early prediction applications. Wang et al. [20] and Liu et al. [31] did not report the accuracy of their models, making direct performance comparisons difficult. Ye et al. [32] developed an ultrasound-dependent model, which might be impractical for routine clinical use. Liu et al. [30] introduced a highly complex ML approach that, while effective, may not be feasible for widespread adoption in standard medical practice.

The present study aimed to overcome the limitations of existing macrosomia prediction models by developing an ML-based approach that relies solely on maternal sociodemographic and obstetric data, eliminating the need for biochemical markers or ultrasound measurements. This design makes our model more accessible for diverse clinical settings, including low-resource environments where advanced diagnostic tools may not be available.

The predictive models were constructed using variables that can be easily collected during basic obstetric care, even in pre-conceptional assessments. Our findings are also consistent with recent evidence showing that maternal metabolic characteristics, particularly pre-pregnancy BMI and glucose-related parameters, remain among the strongest predictors of fetal macrosomia across different machine learning approaches [28,35] The key predictors included maternal age, gravida, BMI, history of diabetes mellitus, and history of hypertension, while for parous women, previous birth weight was additionally included in the parous model. Importantly, we identified maternal age and BMI as the most significant features for macrosomia prediction across both nulliparous and parous women. These predictors were intentionally selected because they are routinely available during standard antenatal care and have consistently been identified as important clinical risk factors for fetal macrosomia in previous studies. Restricting the models to routinely collected variables may improve their applicability in resource-limited settings, although incorporating longitudinal clinical information may further improve predictive performance.

To build robust predictive models, we evaluated multiple ML algorithms, including the Extra Trees Classifier, AVG Blender, LGBM Classifier, XGB Classifier, Logistic Regression, and Random Forest Classifier. Among these, the XGB Classifier demonstrated the best predictive performance, achieving 80% accuracy and an AUC-ROC of 82.2% in nulliparous women, and 72.7% accuracy with an AUC-ROC of 83.3% in parous women. The study also employed 10-fold cross-validation and an independent test set to enhance the model’s generalizability and robustness. The findings suggest that machine learning approaches have potential for future macrosomia risk stratification; however, substantial further validation is required before any clinical application can be considered.

Despite its strengths, this study has several limitations. The most important limitation is the relatively small sample size available for model development and validation. After stratification into nulliparous and parous groups, the effective sample size for each machine learning model became substantially smaller. Therefore, the reported performance metrics should be interpreted as exploratory rather than definitive estimates of predictive performance. Small datasets increase the risk of overfitting, unstable model coefficients, and variability in feature importance rankings. Consequently, the present findings should be considered proof-of-concept results that require confirmation in larger prospective cohorts before clinical implementation. Second, while our model successfully predicted macrosomia using only sociodemographic and obstetric data, it did not incorporate ultrasound measurements or biochemical markers, which may further improve predictive performance. Future studies could investigate hybrid models integrating these parameters. Third, the absence of longitudinal pregnancy data, such as gestational weight gain trajectories, fetal growth trends, and dietary characteristics, may have limited individualized risk prediction. Regarding internal validity, the study employed a predefined train-test separation strategy together with 10-fold cross-validation within the training dataset to reduce overfitting and obtain more robust performance estimates. Nevertheless, the relatively small sample size limits the precision and stability of model performance metrics. Furthermore, repeated cross-validation, bootstrap validation, and sensitivity analyses across multiple random seeds were not performed; therefore, the robustness of model performance estimates could not be fully assessed. In addition, calibration performance was not evaluated. Since discrimination alone is insufficient for assessing clinical prediction models, future studies should include calibration analyses and decision-curve assessment. Regarding external validity, although internal validation was performed using a predefined training-test split together with 10-fold cross-validation within the training dataset, all participants were recruited from a single tertiary referral center. Consequently, the proposed models were evaluated within a single institutional setting and may not be representative of broader obstetric populations with different demographic and clinical characteristics. Therefore, the generalizability of the findings remains uncertain, and external validation using independent multicenter cohorts is essential to confirm model robustness, reproducibility, and clinical applicability before routine clinical implementation. This recommendation is supported by recent evidence demonstrating that externally validated machine learning models incorporating multicenter validation, calibration assessment, and decision-curve analysis provide more reliable estimates of clinical performance [35]. Future studies should therefore adopt similar validation strategies to improve the robustness, generalizability, and clinical applicability of prediction models.

Although the proposed models demonstrated encouraging predictive performance, they are not currently intended to replace clinical judgment or established obstetric assessment methods. Instead, they should be considered exploratory decision-support tools that may assist clinicians in identifying pregnancies at increased risk of macrosomia. In a potential clinical implementation scenario, the model could be applied during the first antenatal visit using routinely collected maternal characteristics. Pregnancies classified as high risk could undergo closer fetal growth surveillance, more frequent antenatal follow-up, nutritional counseling, and earlier referral to specialist obstetric care when appropriate. Because the present study was exploratory, no clinically validated risk threshold has yet been established, and threshold determination should be addressed in future prospective validation studies. Before clinical implementation, prospective validation, calibration assessment, and evaluation of clinical utility through decision-curve analysis will be necessary. Future studies should evaluate model performance across diverse clinical settings before routine implementation. Integration within electronic health record systems may facilitate real-time risk assessment and support individualized obstetric care in the future.

Furthermore, because the present models were based on static baseline variables, they cannot account for temporal changes such as gestational weight gain, fetal growth trajectories, or evolving metabolic status. Future studies incorporating longitudinal data and dynamic prediction frameworks may improve predictive accuracy and individualized risk assessment.

## 5. Conclusions

In conclusion, our findings suggest that routinely available maternal sociodemographic and obstetric characteristics may be sufficient to develop machine learning-based models for the early prediction of fetal macrosomia without requiring ultrasound measurements or biochemical markers. Although the proposed models demonstrated encouraging predictive performance, the findings should be interpreted cautiously because of the pilot nature of the study and the relatively small sample size. Prospective validation, calibration assessment, and decision-curve analysis will be essential to establish the clinical utility of the proposed models.

## Figures and Tables

**Table 1:** The demographic and obstetric findings of the patients included the study.

		Primiparous			Multiparous		
		+	−	*p*	+	−	*p*
Macrosomia		22 (45.8%)	26 (54.2%)	0.174 ^a^	28 (53.8%)	24 (46.2%)	0.09 ^a^
Age (years) mean + std.dev.		25.23∓5.71	27.03∓5.29	0.161 ^b^	30.61∓5.42	28.92∓4.76	0.242 ^b^
BMI (kg/m^2^) mean + std.dev.		32.62∓3.87	28.65∓4.08	0.001 ^a^	31.73∓4.40	31.52∓3.90	0.833 ^b^
Gravida mean + std.dev.		1.31∓0.89	1.15∓0.37	0.749 ^b^	2.82∓1.06	2.67∓0.76	0.852 ^a^
Smoking habits (*n*, %)	+	1 (4.5%)	0 (0%)	0.272 ^c^	1 (3.6%)	0 (0%)	0.350 ^c^
	−	21 (95.5%)	26 (100%)		27 (96.4%)	24 (100%)	
The history of hypertension (*n*, %)	+	1 (4.5%)	0 (0%)	0.272 ^c^	1 (3.6%)	0 (0%)	0.350 ^c^
	−	21 (95.5%)	26 (100%)		27 (96.4%)	24 (100%)	
The history of diabetes mellitus (*n*, %)	+	7 (31.8%)	0 (%0%)	0.002 ^c^	4 (14.3%)	0 (0%)	0.054 ^c,^*
	−	15 (68.2%)	26 (100%)		24 (85.7%)	24 (100%)	
Previous birth weight > 4000 g (*n*, %)	+	Not applicable			5 (17.9%)	1 (4.2%)	0.123 ^c^
	−				23 (82.1%)	23 (95.8%)	

^a^ Student t test; ^b^ Mann–Whitney U (data is not normal); ^c^ Chi-Square. * For primiparous, the history of diabetes mellitus was defined as gestational diabetes mellitus. BMI: Body Mean Index.

**Table 2:** Prognosis prediction results of different machine learning algorithms.

	Model Training Results					
	Primiparous			Multiparous		
Model Name	ROC-AUC	Accuracy	Interval	ROC-AUC	Accuracy	Interval
Extra Trees Classifier	0.6	0.5	0.19–0.81	0.633	0.545	0.251–0.84
Random Forest Classifier	0.62	0.6	0.296–0.904	0.583	0.545	0.251–0.84
Logistic Regression	0.96	0.7	0.416–0.984	0.5	0.636	0.352–0.921
XGB Classifier	0.82	0.8	0.552–1.0	0.833	0.727	0.464–0.99
LGBM Classifier	0.5	0.5	0.19–0.81	0.5	0.545	0.251–0.84

AVG Blender: Average Blender, LGBM: Light Gradient Boosting Machine, XGB: eXtreme Gradient Boosting.

**Table 3:** Classifier confusion matrix.

	XGB Classifier					
	Primiparous			Multiparous		
Macrosomia	Yes	No	%	Yes	No	%
Yes	4	1	80.0 ^a^	3	2	60.0 ^a^
No	1	4	80.0 ^b^	1	5	83.3 ^b^

XGB: eXtreme Gradient Boosting. ^a^ sensitivity; ^b^ specificity.

## Data Availability

The authors certify that this manuscript reports original clinical trial data. Individual, de-identified participant data that underlie the results reported in this article (text, tables, figures, and appendices) are available from the corresponding author following publication, including the clinical study report and study protocol.
